# Mr. Jenkinson on Solutio Arsenici in Rheumatism

**Published:** 1805-06-01

**Authors:** John Jenkinson

**Affiliations:** Manchester


*To the Editors of the Medical and Physical Journal.*

Gentlemen,

Mr. Whitlam's account, in your last Number, of a case of inveterate chronic rheumatism, relieved by the use of the solutio arsenici, was of course exceedingly gratifying to me; and I wish most heartily, for the sake of those afflicted with that tedious and painful disorder, such of your correspondents as have made trial of the same remedy, would give equal publicity to their experience. What I have seen of its effects, since I published my first account in your Journal, has only tended to confirm what I there advanced; and I have scarcely a doubt remaining, but it will prove a most valuable medicine indeed, even in those states of the disease which we have hitherto been very little capable of relieving.

The lady under Mr. Hardman's care, before referred to, has been repeatedly and effectually relieved from returns of the symptoms, by the use of the solution.

William Daval, a farming labourer in Staffordshire, ætat. 60, in the course of the last summer obtained a complete cure, by the same means, from a combination of lumbago and sciatica, which had crippled him for months, and baffled the force of the usual remedies. I had the pleasure of seeing him about two months after his recovery, holding the plough with his usual strength and spirits. His thankfulness was excessive; and his expression of it was such as would have excited delightful emotions, even in the calmest of beings.

As the safety of the remedy has been established, by a long and general employment of it, in other complaints, I hope the liberal humanity of my professional brethren will prompt them to have recourse to it in chronic rheutism, upon the earliest occasions, and to communicate the result of their experience without unnecessary delay; in all probability, the comforts, if not the existence of many fellow creatures, now miserable, are depending upon their compliance. I am, &c.

*Manchester, Jan.* 12, 1805. JOHN JENKINSON.

